# Overview of a Knowledge Translation (KT) Project to improve the vaccination experience at school: The CARD™ System

**DOI:** 10.1093/pch/pxz025

**Published:** 2019-03-29

**Authors:** Anna Taddio, C Meghan McMurtry, Lucie M Bucci, Noni MacDonald, Anthony N T Ilersich, Angelo L T Ilersich, Angela Alfieri-Maiolo, Christene deVlaming-Kot, Leslie Alderman, Pain Pain Go Away Team, Tamlyn Freedman, Tori McDowall, Horace Wong, Kate Robson, Christine Halpert, Evelyn Wilson, Jocelyn Cortes, M Mustafa Hirji, Cathryn Schmidt, Srdjana Filipovic, Melanie Badali

**Affiliations:** 1 Leslie Dan Faculty of Pharmacy, University of Toronto, Toronto, Ontario; 2 The Hospital for Sick Children, Toronto, Ontario; 3 University of Guelph, Guelph, Ontario; 4 Immunize Canada, Ottawa, Ontario; 5 Dalhousie University, Halifax, Nova Scotia; 6 Niagara Region Public Health & Emergency Services, Thorold, Ontario; 17 Canadian Family Advisory Network; 18 British Columbia Centre for Disease Control; 19 Ontario Institute for Studies in Education, University of Toronto; 20 Ontario Ministry of Health and Long-Term Care; 21 AnxietyBC, Vancouver, British Columbia

**Keywords:** Vaccination, Pain management, Knowledge translation

## Abstract

**Background:**

Students experience fear, pain, and fainting during vaccinations at school. While evidence-based interventions exist, no Knowledge Translation (KT) interventions have been developed to mitigate these symptoms. A multidisciplinary team—the Pain Pain Go Away Team—was assembled to address this knowledge-to-care gap. This manuscript provides an overview of the methodology, knowledge products, and impact of an evidence-based KT program developed and implemented to improve the vaccination experience at school.

**Methods:**

We adapted knowledge and assessed the barriers to knowledge use via focus group interviews with key stakeholder groups involved in school-based vaccinations: students, nurses, school staff, and parents. Next, we developed project-specific goals and data collection tools and collected baseline data. We then created a multifaceted KT intervention called The CARD™ System (C-Comfort, A-Ask, R-Relax, D-Distract) to provide a framework for planning and delivering vaccinations using a student-centred approach. Selected KT tools from this framework were reviewed in additional focus groups held in all stakeholder groups. The multifaceted KT intervention was then finalized and implemented in stages in two projects including grade 7 students undergoing school vaccinations and impact on student outcomes (e.g., symptoms of fear, pain, dizziness) and process outcomes (e.g., utilization of interventions that reduce student symptoms, vaccination rate) were assessed.

**Results:**

Participants reported that improving the vaccination experience is important. Based on participant feedback, an evidence-based multifaceted KT intervention called The CARD™ System was developed that addresses user needs and preferences. Selected KT tools of this intervention were demonstrated to be acceptable and to improve knowledge and attitudes about vaccination in the stakeholder groups. In two separate implementation projects, CARD™ helped grade 7 students prepare for vaccinations and positively impacted on their vaccination experiences. CARD™ improved vaccination experiences for other stakeholder groups as well. There was no evidence of an impact on school vaccination rates.

**Conclusion:**

We developed and implemented a promising multifaceted KT intervention called The CARD™ System to address vaccination-associated pain, fear, and fainting. Future research is recommended to determine impact in students of different ages and in different geographical regions and clinical contexts.

School-based vaccination programs are an efficient way to deliver vaccinations to youth (1). Despite the effectiveness of this venue for vaccinating large numbers of school-aged children, many youth have negative experiences with school vaccinations due to concerns about injection-related pain (2,3). Fear of pain and needles can lead to an increase in pain perception, fainting, and procedure refusal (4). Negative attitudes and experiences can lead to future vaccination hesitancy, noncompliance with vaccination and noncompliance with other health care interventions (4). The Ontario Ministry of Health and Long-term Care’s plan to modernize Ontario’s immunization system (‘Immunization 2020’) (5) as well as the 2014 Annual Report of the Chief Medical Officer of Health of Ontario (‘Vaccines: the best medicine’) (6) specifically identify pain reduction as a key strategic step to an effective immunization system for the province. The World Health Organization also recommends addressing pain mitigation in the school setting (7).

In 2015, we undertook a systematic review of the research evidence for interventions to reduce vaccination-related pain, fear, and fainting. This systematic review served as the evidence base for a Clinical Practice Guideline (CPG) on this topic (8). Knowledge Translation (KT) tools for incorporating the CPG recommendations in the school vaccination context could not be included due to a gap in the evidence base for this practice setting.

The school vaccination setting is complex and involves the interplay of multiple stakeholders that may influence intervention delivery and effectiveness, including health providers, students, school staff, and parents. Individual practitioners are limited in their ability to make changes to how pain and fear are handled in students without involving the other stakeholder groups. Change is required at both the individual health care provider level as well as the system level to address all the potential barriers to best practices (9).

Selected members of the CPG panel partnered with a public health unit (Niagara Region Public Health) and school board (Niagara Catholic District School Board) to undertake a program of research aimed at developing a multifaceted KT intervention tailoring the CPG recommendations for the school setting. The aim was to improve the vaccination experience at school. This article is one in a series of 6 that describe this work (10). The purpose of this article is to provide an overview of the steps involved in the project, key findings, and to serve as the repository for the key tools that have been created. The remaining articles in the series provide more detail regarding the various project steps and findings.

## METHODS

### Conceptual framework

The project was guided by the Knowledge to Action (KTA) (11) cycle and the Consolidated Framework for Implementation Research (CFIR) (12). The KTA (11) cycle articulates the translation of research evidence into practice as the interplay between knowledge creation and action. CFIR (12) specifies a list of constructs that positively and negatively influence implementation (e.g., intervention characteristics) and can be used to guide and assess implementation of interventions. An integrated KT approach was used, involving all stakeholders throughout and tailoring knowledge to meet their needs (13).

### Pain Pain Go Away Project Team

A multidisciplinary, multi-sectoral group of individuals, the Pain Pain Go Away Team, oversaw the project. The team included 20 members: 3 clinician-scientists (pharmacy-AT, psychology-CMM, medicine-NM) with content expertise in vaccination, pain, fear, and fainting mitigation; 2 clinicians (regional public health school nurse-TM, psychologist-MB); 3 regional public health unit managers (clinical services-AAM, school programs-CdVK, vaccine preventable disease program-LA); 2 policy makers (regional public health unit associate medical officer of health-MMH, provincial ministry of health representative-JC); 1 parent advocate (KR); 2 students (13 and 17 years old-ALTI, ANTI); 2 educators (school educator-EW, public health-CH); 2 KT experts (vaccination promotion-LMB, hospital quality improvement-SF); 1 multimedia producer (CS), and 2 graduate trainees (TF, HW).

Monthly or bimonthly meetings were held with the group to discuss progress of the project and to plan next steps. In addition, three subgroups were created to oversee specific project components: 1) project management, including data collection and analysis; 2) development of the multifaceted KT intervention; and 3) implementation planning and execution. The lead scientist (AT) oversaw the project. Ethical approval was granted by the Research Ethics Board of the University of Toronto.

### Step 1: Identifying potential areas for intervention and published guidelines

In previous work within the Knowledge Creation cycle of the KTA (11), we undertook primary studies to identify student perceptions of school vaccinations, analgesic practices, and the impact of pain and fear on vaccine acceptance (2,3,14,15). These studies demonstrated that: 1) fear of injection-related pain is prevalent in students; 2) interventions to mitigate fear and pain are under-prioritized and suboptimally utilized; and 3) concerns about needle-related pain contribute to vaccine refusal. We carried out a knowledge synthesis and developed a CPG (8) with recommendations for reducing pain, fear and fainting during vaccination. Template tools were created to assist clinicians with implementation of the recommendations; however, they were not specific to the school vaccination context.

### Step 2: Adapt knowledge to local context and assess barriers to knowledge use

We tackled the Action cycle of the KTA framework and used a multicomponent strategy to develop tools and processes for the local school vaccination context. This included: 1) focus groups with stakeholders to learn about their experiences and obtain feedback on template tools; 2) determination of what outcomes to measure and the manner of their assessment; and 3) examination of current policies and practices.

We identified interventions from our CPG (8) that could be adapted for the school vaccination context and created template KT tools. We then carried out focus group interviews with four different types of stakeholders: students, parents, school staff and public health nurses. Within each focus group, participants were asked to share experiences with school vaccinations, strategies used, as well as challenges and facilitators of a positive vaccination experience. Participants were also asked to provide detailed oral and written feedback on template KT tools and implementation strategies. These focus groups provided us with key themes regarding the barriers and facilitators faced in daily practice related to pain and fear management specific to the Niagara context (16). A cause-and-effect (fishbone) diagram was developed to describe current practice (Figure 1).

**Figure:**
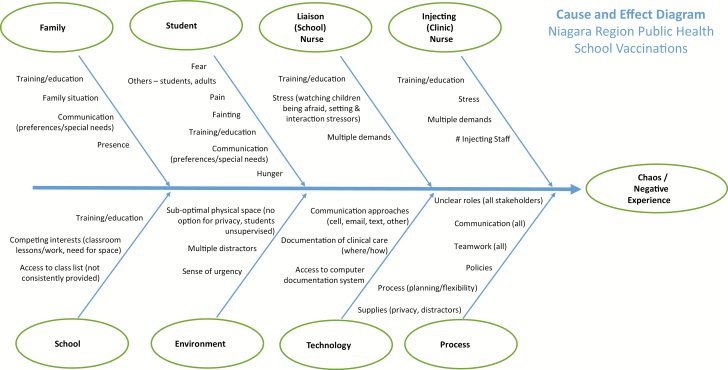
Figure 1. The figure is also available online as a full-sized, downloadable resource.

A concurrent separate activity involved identification of the project outcome indicators and monitoring tools by the project team. First, a list of prioritized outcomes was created based on our CPG (8) (Table 1). Then monitoring tools were refined or developed to track these outcomes (Supplementary Appendices 1–6) and included: 1) tool feedback survey; 2) knowledge survey; 3) student vaccination symptom survey (i.e., pain, fear, and dizziness-precursor of fainting); 4) nurse vaccination intervention documentation checklist; 5) intervention fidelity checklist; and 6) process checklist. Qualitative methods (informal feedback/debriefs, focus group interviews) were selected as the approaches to evaluate satisfaction and supplement quantitative data.

**Table 1.** Outcome indicators for the project

Category	Measures
Clinical/patient	Student fear, pain, dizziness (precursor of fainting), fainting, and returns to clinic because feeling unwell
Clinic Process	Flow of events/workflow during vaccination, utilization of interventions, vaccine compliance/procedure success
Acceptability (students, parents, school staff, nurses)	Understandability, quantity, and quality of education
Satisfaction (students, parents, school staff, nurses)	Vaccination experience, value, and effectiveness of education
Attitudes (any stakeholder group)	Attitudes about pain, fear, and vaccination
Knowledge (any stakeholder group)	Knowledge about effective interventions for pain, fear, and fainting
Competence	Health provider education; skill competency

An audit was conducted to benchmark current practices and included documentation of pain and fear interventions used during vaccinations and student symptom scores. From these audits, the group identified goals for improvement. Separately, existing policies and processes of delivery in the school-based vaccine program were reviewed by the implementation team to examine alignment with identified needs, preferences, and opportunities for change.

### Step 3: Selecting and tailoring intervention tools and processes for the local context

The results from Step 2 coupled with our CPG recommendations (8) were used to inform the development of an evidence-based multifaceted KT intervention called The CARD™ System (C-Comfort, A-Ask, R-Relax, D-Distract). CARD™ provides a framework for planning and delivering vaccinations using a student-centered approach. Each letter of the word (i.e., C, A, R, and D) represents a different category of interventions that can help guide planning and delivery of vaccinations in order to optimize the student experience and coping. Important vaccination planning activities include: 1) securing appropriate spaces for vaccination clinics such as the school library, 2) confirming that these spaces are available and that individuals are aware of upcoming vaccination days, 3) educating students using CARD™ educational resources, and 4) having students select their preferred coping strategies using the student CARD™ pamphlet. Important vaccination day activities include: 1) setting up the clinic to minimize visual cues that promote fear and spreading of fear to others, 2) visiting the classroom to introduce clinic staff and remind students of CARD™, 3) identifying and triaging students with fear and special requests, and 4) using CARD™ during interactions with students.

On vaccination day, nurses explicitly ask students about their level of fear and what ‘*CARDs they want to play*’ to help them cope. They then support students in their choices. With CARD™, students are invited to actively participate in their health care and *play*/select specific strategies within the four different letter categories according to their preferences. For example, a student may choose to play an ‘A’ and *A*sk to be vaccinated in private rather than in front of their peers and/or ‘D’ and bring an electronic device to serve as a *D*istraction. Afterwards, students are asked about their vaccination symptoms (i.e., fear, pain, dizziness). Importantly, with CARD™, bundling of interventions is possible in that students can play *multiple CARDs at the same time*. CARD™ allows students to take charge of their pain and fear and choose interventions that meet their individual needs.

Two student team members were integral to development of this KT intervention and associated resources. The role of each of them will be briefly described. The first one (ANTI, 17 years old) created the name CARD™ to capture the principles of the KT intervention in an engaging and intuitive framework for users. Both students were involved in creating two videos that addressed student-prioritized educational gaps (i.e., procedural preparation and coping with pain, fear, and fainting) (16). The first video (4 minutes) (https://youtu.be/z57vTpb19wQ) provides basic information about vaccines; this video instructs students on what a vaccine is and how it works, side effects of vaccines, and the process for school-based vaccinations, including consent and what will happen on the day of vaccination. The second video (7 minutes) (https://youtu.be/c41HvgEKQSk) instructs students in the CARD™ mnemonic. Vignettes of students undergoing vaccination with demonstrations of the different interventions are included. Both student team members scripted the videos and the second student team member (ALTI, 13 years old) narrated them.

Three separate companion pamphlets were developed to complement the videos for students, school staff, and parents, respectively (Figures 2–4). A poster was also created for schools (Figure 5). The student CARD™ pamphlet includes examples of strategies for each letter of the word and fill-in-the-blank spaces so that students can record the interventions they want to use for their upcoming vaccination. The parent and school staff pamphlets include information regarding vaccination and CARD™. Of note, videos and pamphlets were selected as the primary delivery methods for education of stakeholders to comply with preferences as well as maximize the standardization of messaging and enhance portability (e.g., feasible access across settings).

**Figure:**
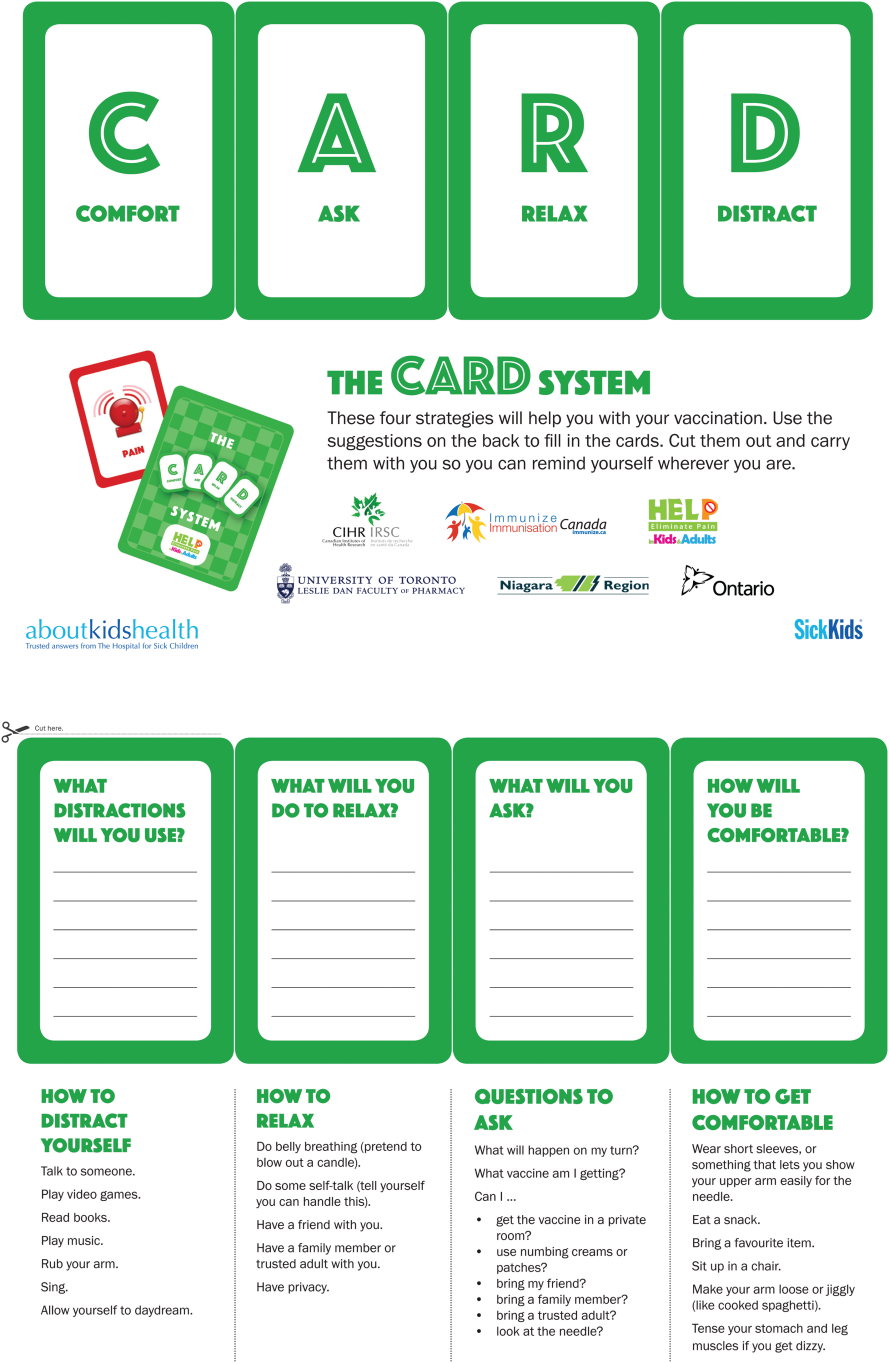
Figure 2. The figure is also available online as a full-sized, downloadable resource.

**Figure:**
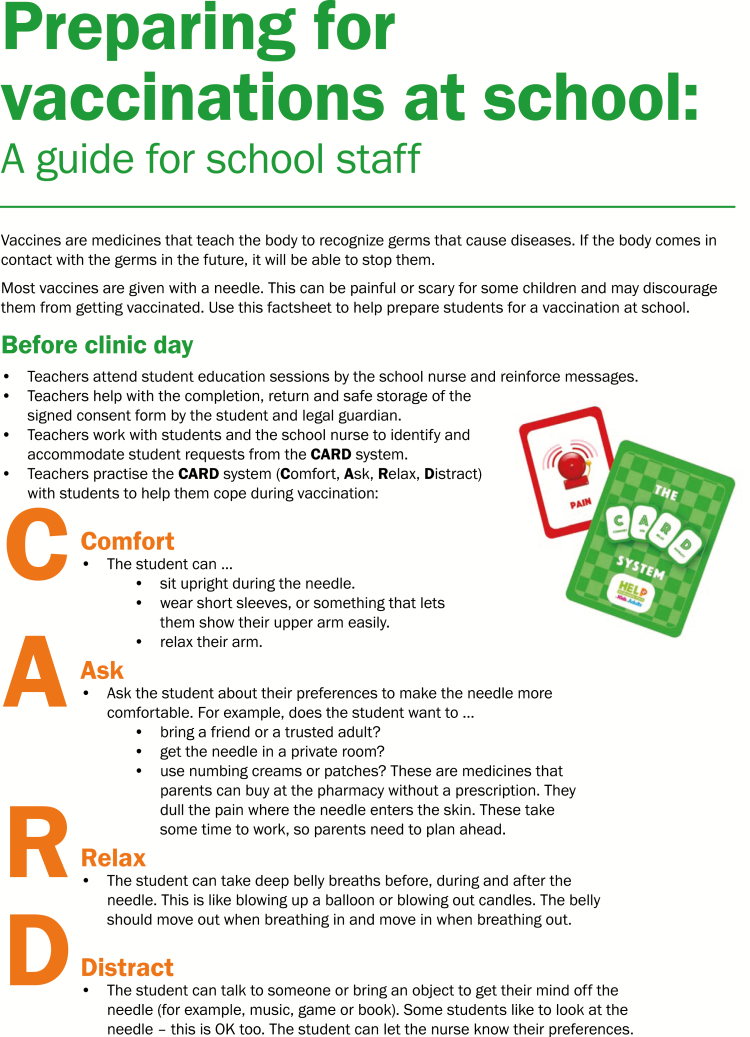
Figure 3a. The figure is also available online as a full-sized, downloadable resource.

**Figure:**
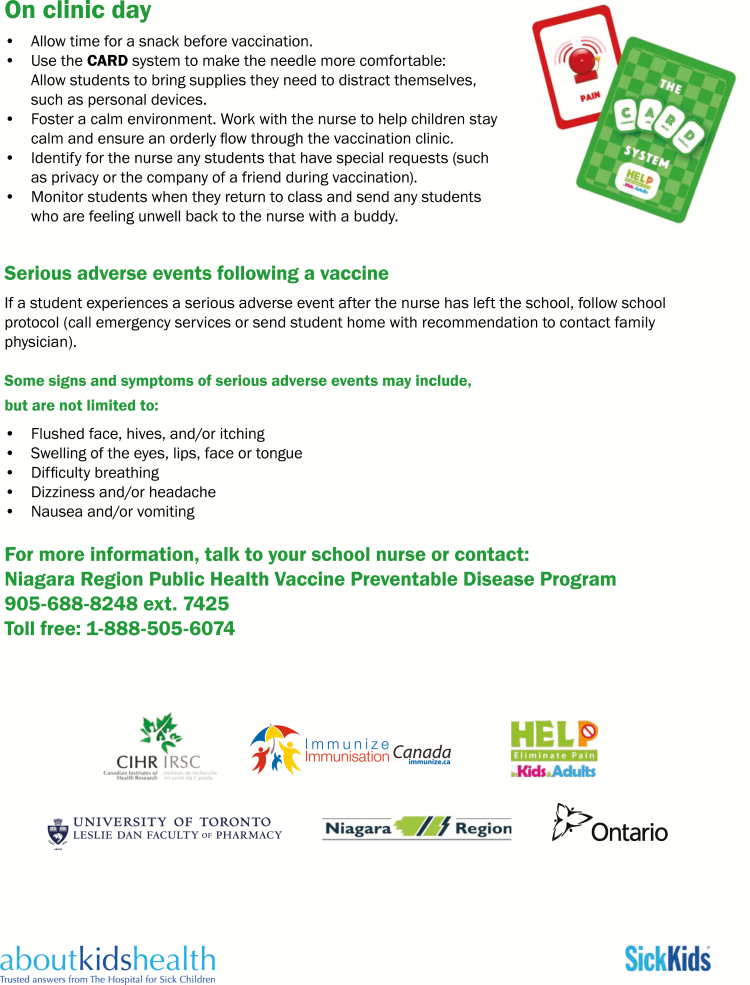
Figure 3b. The figure is also available online as a full-sized, downloadable resource.

**Figure:**
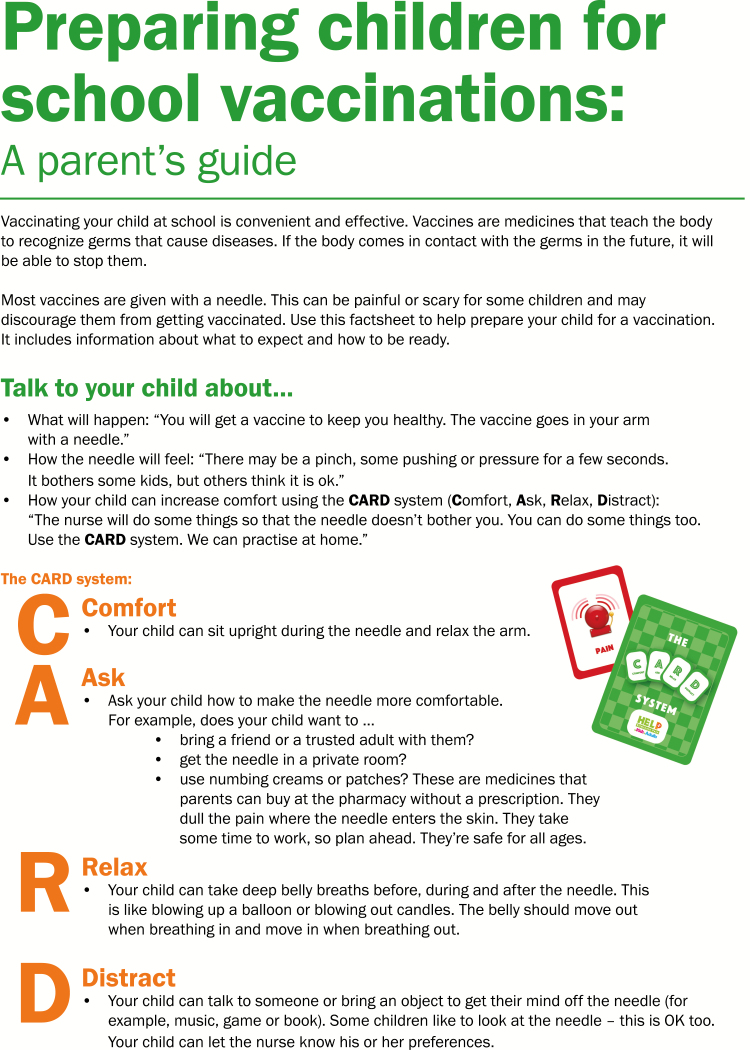
Figure 4a. The figure is also available online as a full-sized, downloadable resource.

**Figure:**
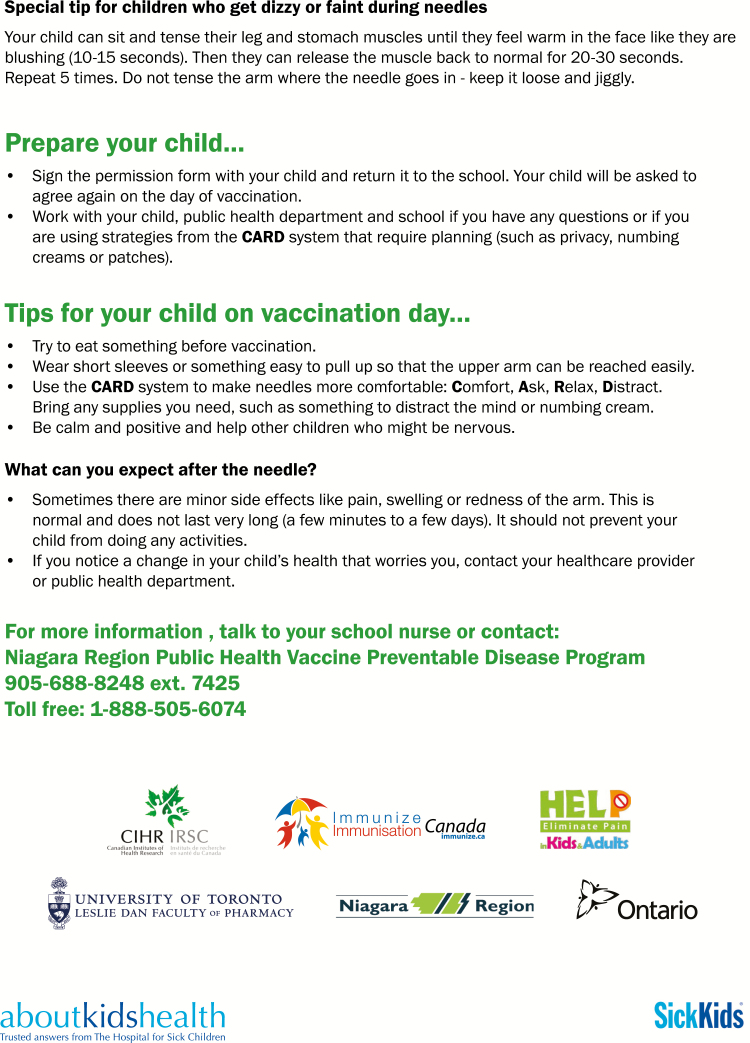
Figure 4b. The figure is also available online as a full-sized, downloadable resource.

**Figure:**
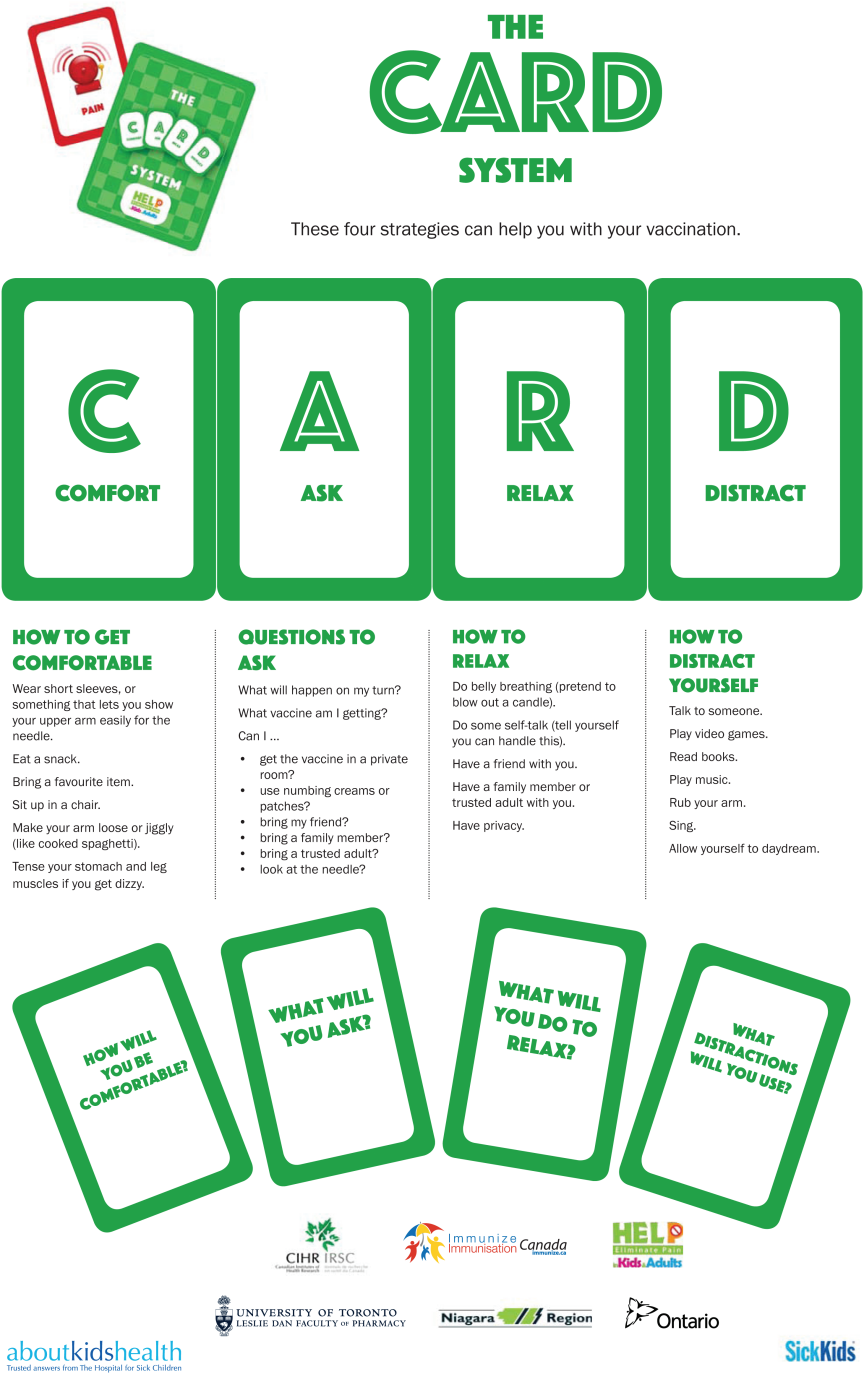
Figure 5. The figure is also available online as a full-sized, downloadable resource.

A toolkit was created for The CARD™ System to support implementation. Identified opportunities for integration and alterations in existing workflow processes and activities were discussed and a preliminary implementation plan was proposed. A summary of components of the toolkit, targeted stakeholder group, and time of implementation relative to vaccination are described in Table 2. Of note, one of the included tools is a template communication and planning checklist to be used by nurses in clinic planning and execution that incorporates The CARD™ System (Figure 6).

**Table 2.** Components of the multifaceted Knowledge Translation (KT) intervention (The CARD™ System)

CARD™ resources	Description of resource	Implementation Prior to vaccination	Implementation On vaccination day
Video 1 – What you need to know about vaccines at school	4-min video describing vaccination and the process for school vaccination clinics. This video is shown to students by school nurse* at school during a classroom lesson (https://youtu.be/z57vTpb19wQ)	+	−
Video 2 – The CARD™ System: Play your power CARD™	7-min video describing CARD™ with vignettes of students demonstrating the different interventions. This video is shown to students by school nurse* at school during a classroom lesson (https://youtu.be/c41HvgEKQSk)	+	−
Slide presentation	Overview of vaccines offered during school clinics and practice case scenarios for CARD™ reviewed with students by school nurse* at school during a classroom lesson	+	−
Factsheets for students	CARD™ pamphlet with fill-in-the blank space for students to record preferred interventions. This pamphlet is reviewed with students by school nurse* at school during classroom teaching and is used for clinic planning (Figure 2)	+	+ /−
Factsheets for school staff	CARD™ and vaccine process pamphlet given by school nurse* to teachers and other school staff (Figure 3)	+	−
Posters for school	CARD™ poster given by school nurse* to teacher for classroom (Figure 5)	+	+
Factsheets for parents	CARD™ and vaccine process pamphlet given to students by school nurse* to bring home with vaccine consent forms (Figure 4)	+	−
Point of care tool for nurses	Communication and planning checklists for pre-vaccination day (e.g., securing a private space, permission for use of personal electronic devices, vaccination day reminders) and vaccination day (e.g., separate waiting and vaccination area, triaging students, using CARD™ during vaccination) activities to be used by school nurse* and injecting nurse (Figure 6)	+	+
Assessment and management	Assessment of student level of fear prior to vaccination and implementation of student-selected CARD™ strategies during vaccination by injecting nurse	−	+
Table poster/divider	Table poster/divider with picture of the word ‘CARD’ to obstruct needle preparation by injecting nurses and serve as cue to students and injecting nurses to discuss and use CARD™	−	+
Distraction toolkits	Distraction toolkits for all vaccine clinic workstations – contents include spinners, bubble pens, pipe cleaners	−	+
Presence of school nurse	School nurse* presence at all vaccine clinics (familiar face for students and school staff); assist with clinic flow, support students and injecting nurses, liaise with school staff	−	+
Audit and Feedback from vaccine clinics	Student symptom survey (pain, fear, dizziness-precursor of fainting) (Supplementary Appendix 3); Injecting nurse checklist of interventions used, number of injections administered (Supplementary Appendix 4) Process issues documentation checklist, including number of students returning to clinic because feeling unwell (Supplementary Appendix 6)	−	+
Internal Champions	School nurse* and injecting nurse assigned to study to network with team members to promote best practices, answer project questions, liaise with managers regarding project	+	+
Training material resources for front-line public health staff	Resource Binder used for training session with school nurses* and injecting nurses. Includes: scientific evidence, alignment with organization mission/values, policies and work processes, video links, slide presentation, point of care tools, pamphlets, case scenarios, contact information of project champions, certificate of attendance	+	−
Video 3 – Improving the vaccination experience at school **	12-min training video for public health and school staff describing CARD™, including; planning and vaccination day activities with vignettes of students undergoing vaccination and testimonials (https://youtu.be/FXj6ELi4BVg)	+	−

+ = Yes; – = No.

*School nurses are nurses that are assigned to individual schools. They are familiar with the physical layout of the schools and have a working relationship with school staff and students. They typically organize and attend the first vaccination clinic. Some public health units may not have a school nurse and other individuals would carry out these activities.

**This video was created at the end of the project to support future training and implementation.

**Figure:**
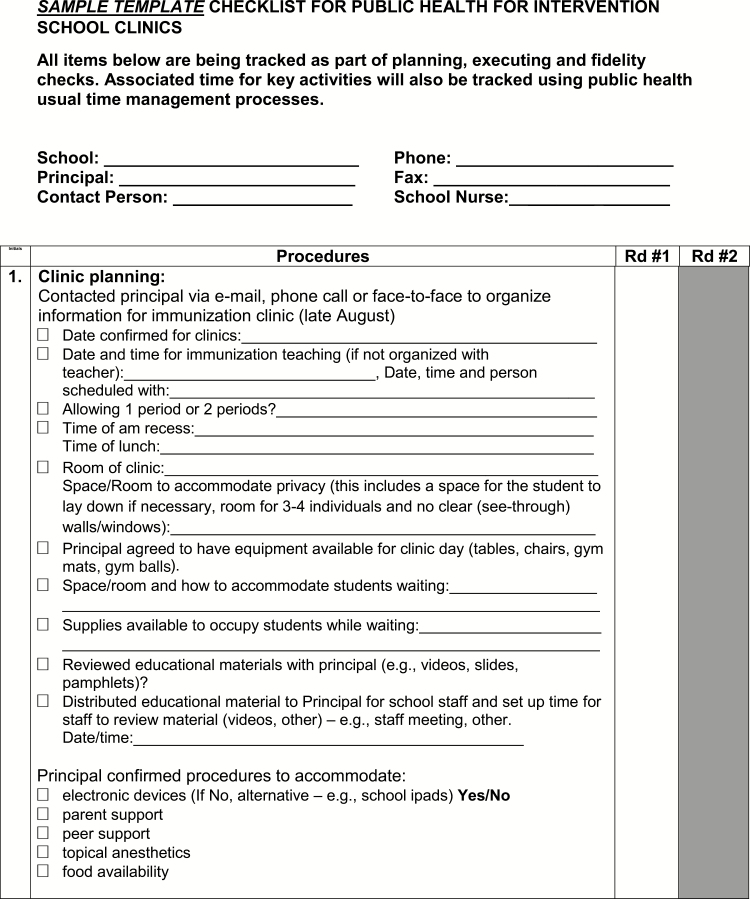
Figure 6a. The figure is also available online as a full-sized, downloadable resource.

**Figure:**
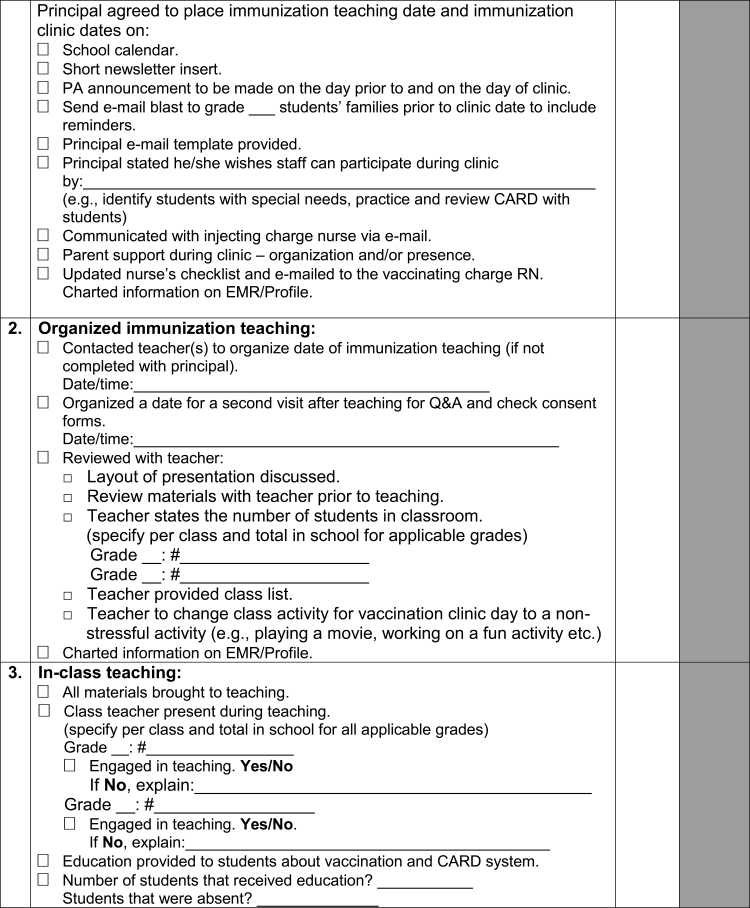
Figure 6b. The figure is also available online as a full-sized, downloadable resource.

**Figure:**
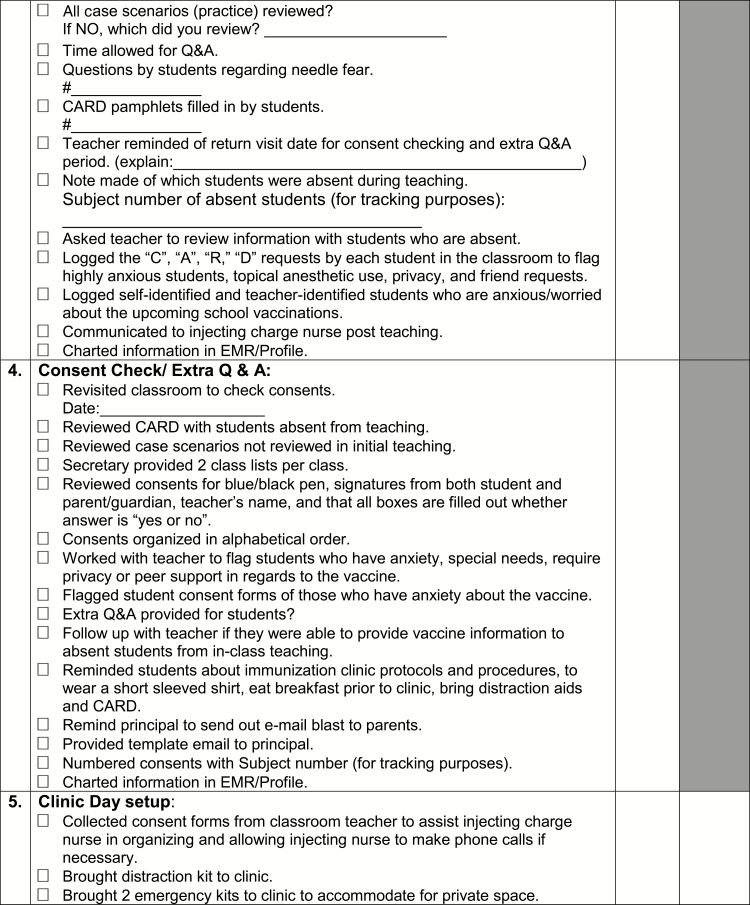
Figure 6c. The figure is also available online as a full-sized, downloadable resource.

**Figure:**
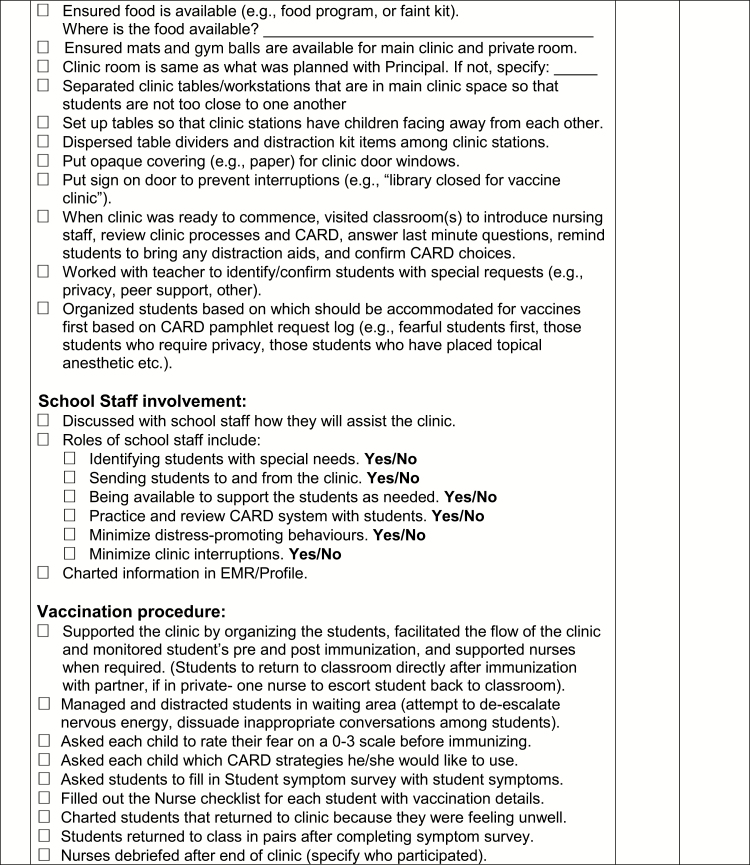
Figure 6d. The figure is also available online as a full-sized, downloadable resource.

With respect to implementation timing, it is important to note that in many jurisdictions, public health nurses already routinely visit schools to plan vaccination clinics, educate students about vaccination and distribute consent forms; this typically occurs 1-4 weeks prior to vaccination clinics. The current standard of practice in Niagara Region Public Health consists of vaccine education that focuses on information about the diseases and vaccines. Guided by student learning needs identified in extant literature and the results from Steps 1 and 2, we proposed reducing the amount of time allocated to diseases and vaccines and adding information about what will happen during vaccination (procedural information) and how to cope (pain, fear, and fainting mitigation strategies). On vaccination day, proposed changes focused on planning for a suitable clinic environment and processes, including; minimizing visual cues that elicit fear, and implementing student-directed interventions to minimize pain, fear, and fainting.

Within the Action cycle of the KTA framework, the next step consisted of finalizing the multifaceted KT intervention and implementation plan. Focus group interviews were repeated with all stakeholder groups to solicit feedback about the key KT tools (two videos, three pamphlets) and implementation approaches (17). Feedback was overwhelmingly positive. In addition, evaluation of the impact of the KT tools on conceptual knowledge and attitudes about fear and pain demonstrated significant improvements post review of the KT tools. Minor edits were made to the videos and pamphlets to address feedback.

Discussions were held with the implementation planning and execution team to identify priorities and create an action plan. Informed by the fishbone diagram, existing policies and work processes were collaboratively reviewed and altered to align with the proposed changes. Then, the implementation plan and KT tools were presented to the entire project team and approved. An educational workshop was prepared to train relevant front-line public health unit staff (i.e., injecting [and charge] nurses, school liaison nurses) involved in school vaccinations. Managers and researchers were present and delivered aspects of the program. The training included review of: rationale for the project, scientific evidence, alignment with organizational values/mission, relevant policies and work processes, videos, pamphlets, and point of care resources. Detailed case scenarios were incorporated into the training to allow for discussion and practice. During the training, nurses provided additional comments (captured in focus groups) (17) and then the implementation plan was finalized.

### Step 4: Implementing interventions and monitoring knowledge use and outcomes

The next step in the Action cycle included executing the multifaceted KT intervention to promote awareness and uptake of the interventions during school-based vaccinations. We rolled out the implementation in two phases. In the initial phase, we undertook a limited implementation of CARD™ whereby we showed the two videos and student pamphlet (Figure 2) to some grade 7 students in a Toronto school in a focus group prior to and after their school vaccinations and monitored knowledge use, acceptability, and impact on the vaccination experience (18).

In the second phase, we implemented the entire multifaceted KT intervention program (The CARD™ System) for grade 7 vaccinations in a controlled clinical trial involving 10 Niagara region schools (5 CARD intervention schools and 5 control schools without any changes to usual care) and evaluated impact on all prioritized outcomes. We demonstrated improvements in student symptoms (e.g., fear, dizziness) and increased utilization of interventions to reduce student symptoms (e.g., distractions, peer support). There was satisfaction with the KT intervention and support for continuing it beyond the project. There was no evidence of an impact on vaccination rate (19,20).

Postimplementation feedback led to the creation of a separate 12-minute educational video after the completion of the project targeted to public health and school staff about CARD™ (https://youtu.be/FXj6ELi4BVg). In it, the different elements of CARD™ are reviewed and demonstrated, including vignettes of students undergoing vaccination using the CARD™ approach. This video is intended to be used as an additional resource to support CARD™ training and implementation.

## SUMMARY

Our 2015 CPG provides recommendations for reducing pain, fear, and fainting associated with vaccine injections (8); however, included KT tools do not address how to implement the recommendations in school-based vaccination clinics. Guided by the KTA (11) and CFIR (12) frameworks, we used an integrated KT approach (13) and worked with the relevant stakeholders to identify ways to embed the CPG (8) into school-based vaccination programs.

In this manuscript, we provided an overview of the project and described our approach to creating a multifaceted KT intervention (The CARD™ System) that translates our CPG (8) recommendations to the school vaccination context. This involved adapting knowledge to the local context, assessing barriers to knowledge use, selecting, tailoring and implementing interventions, and evaluating knowledge use and impact on the vaccination experience and other vaccination program delivery outcomes.

The CARD™ System is a framework for planning and delivering vaccinations that promotes student-centred care and coping. CARD™ integrates procedural information and a simple mnemonic to teach students about how to cope with pain, fear and fainting during vaccination. Key tools from this project are being shared to facilitate uptake more broadly (Table 2, Supplementary Appendix Figures). While developed within the southern Ontario context, intervention components are transferable to other health units and settings. The two videos, for example, can be shown to students in classrooms without public health nurses present (as was done in our first implementation project; described in a subsequent article in this series) (18). When arranging for clinics, nurses can ensure that physical spaces and processes are used that are conducive to reducing fear, including; minimizing visually fearful cues, securing a private space for students that do not wish to be vaccinated in front of peers, and enabling students to use distractions. On the day of vaccination, injecting nurses can bring table top posters/dividers and distraction agents. They can ask students about their level of fear immediately before vaccination and use the language of CARD™ to interact with and coach them during vaccination. The appealing language facilitates communication among students and adults and enables all to become engaged partners in the pain management process (21). Addressing student concerns about pain and fear also demonstrates to them that nurses care and contribute to building trusting relationships (22).

Even if students are not vaccinated at school, they can benefit from education about CARD™. They learn skills for coping with pain, fear and fainting. They also learn how to support others, including their peers or siblings, who are being vaccinated at school. It is important to note that CARD™ is not specifically intended for students with needle phobia; these individuals typically require the expertise of providers trained in anxiety disorders (e.g., psychologists) before they can undergo vaccine injections.

Some additional resources are required to deliver CARD™, primarily related to personnel time allocated for vaccination planning (securing adequate spaces and conditions, education of students, planning for student requests) and photocopying CARD™ pamphlets for students. We note, however, that after the project was completed, Niagara Region Public Health adopted CARD™ across the entire school vaccination program, including approximately 150 schools, without any commensurate changes to staffing levels.

Involvement of external stakeholders is highly recommended to optimize implementation success. To this end, we suggest that public health units providing school vaccination services review their current processes and work with the different stakeholders in their communities, particularly school staff, to determine how best to incorporate these recommendations in their school vaccination programs to improve the vaccination experience at school. School staff can assist with delivery of the education (e.g., if public health nurses are not present in the school and/or students are absent during public health classroom lessons) and reinforce learning.

Finally, it is important to note that this multifaceted KT intervention is consistent with accepted frameworks for health care (patient-centred care, UNICEF’s ladder of participation) (23,24) and education (25) that call for student involvement. It also addresses students’ most pressing concern about vaccination—*the needle*. Students learn to manage fear and pain which represent important life skills.

In the next five papers in this series, we describe the details of the development and testing of this multifaceted KT intervention for the school vaccination setting (16–20). By sharing the processes, key tools and findings from this project, we hope to inform others looking for an evidence-based KT intervention to improve vaccination delivery with a model to use. CARD™ can be tailored to children of different ages and across geographical and medical settings where vaccinations (and other needle procedures) are undertaken. Individuals and organizations wishing to customize CARD™ for their own setting, including the interventions included in the different categories, are encouraged to contact investigators for additional information and to use tools (see also aboutkidshealth.ca/CARD).

## Supplementary Material

Supplementary Figure 1

Supplementary Figure 2

Supplementary Figure 3

Supplementary Figure 4

Supplementary Figure 5

Supplementary Figure 6

Supplementary Appendix 1

Supplementary Appendix 2

Supplementary Appendix 3

Supplementary Appendix 4

Supplementary Appendix 5

Supplementary Appendix 6

## Notes

*Funding Information*: This project was funded by the Canadian Institutes of Health Research Knowledge to Action Grant (KAL-147564).

*Potential Conflicts of Interest:* AT reports Section 9 Trademark No. 924835 for CARD. LMB reports that Immunize Canada received grants from Pfizer Canada, Merck Canada, GSK Canada, Seqirus Canada and Sanofi Pasteur outside the submitted work. There are no other disclosures. All authors have submitted the ICMJE Form for Disclosure of Potential Conflicts of Interest. Conflicts that the editors consider relevant to the content of the manuscript have been disclosed.

*Pain Pain Go Away Team*: Tamlyn Freedman BAS^1^, Tori McDowall RN^6^, Horace Wong MSc^1^, Kate Robson^7^, Christine Halpert RN MA^8^, Evelyn Wilson MAEd BPHE^9^, Jocelyn Cortes RN^10^, M. Mustafa Hirji MD MPH^6^, Cathryn Schmidt^2^, Srdjana Filipovic PhD^2^, Melanie Badali PhD^11^

^1^Leslie Dan Faculty of Pharmacy, University of Toronto, Toronto, Ontario; ^2^The Hospital for Sick Children, Toronto, Ontario; ^6^Niagara Region Public Health & Emergency Services, Thorold, Ontario; ^7^Canadian Family Advisory Network; ^8^British Columbia Centre for Disease Control; ^9^Ontario Institute for Studies in Education, University of Toronto; ^10^Ontario Ministry of Health and Long-Term Care; ^11^AnxietyBC, Vancouver, British Columbia

## References

[1] Vandelaer J, Olaniran M Using a school-based approach to deliver immunization—global update. Vaccine 2015;33(5):719–25. 25523525 10.1016/j.vaccine.2014.11.037

[2] Taddio A, Ilersich AF, Ilersich AN, Wells J From the mouth of babes: Getting vaccinated doesn’t have to hurt. Can J Infect Dis Med Microbiol 2014;25(4):196–200. 25285123 10.1155/2014/470261 PMC4173939

[3] Bucci LM, MacDonald NE, Sondagar C, Taddio A Taking the sting out of school-based immunizations. Paediatr Child Health 2017;22(1):41–2. 29483793 10.1093/pch/pxx004 PMC5819849

[4] McMurtry CM, Pillai Riddell R, Taddio A, et al; HELPinKids&Adults Team Far from “just a poke”: Common painful needle procedures and the development of needle fear. Clin J Pain 2015;31(10 Suppl):S3–11. 26352920 10.1097/AJP.0000000000000272 PMC4900413

[5] Ontario Ministry of Health and Long-Term Care. Immunization 20/20: Modernizing Ontario’s publicly funded immunization program December, 2015. <http://www.health.gov.on.ca/en/common/ministry/publications/reports/immunization_2020/immunization_2020_report.pdf> (Accessed January 19, 2019).

[6] Ontario Ministry of Health and Long-Term Care. Vaccines: the best medicine – 2014 Annual report of the Chief Medical Officer of Health March, 2016 <http://www.health.gov.on.ca/en/common/ministry/publications/reports/cmoh_14_vaccines/default.aspx> (Accessed January 19, 2019).

[7] World Health Organization. Reducing pain at the time of vaccination: WHO position paper – September 2015. WER 2015;90 (No. 39):505–516. <http://www.who.int/wer/2015/wer9039.pdf?ua=1> (Accessed November 14, 2017). 26410893

[8] Taddio A, McMurtry CM, Shah V, et al; HELPinKids&Adults Reducing pain during vaccine injections: Clinical practice guideline. CMAJ 2015;187(13):975–82. 26303247 10.1503/cmaj.150391 PMC4577344

[9] Contandriopoulos D, Lemire M, Denis JL, Tremblay E Knowledge exchange processes in organizations and policy arenas: A narrative systematic review of the literature. Milbank Q 2010;88(4):444–83. 21166865 10.1111/j.1468-0009.2010.00608.x PMC3037172

[10] Taddio A Pain Pain Go Away: Improving the vaccination experience at school. Paediatr Child Health 2019. doi:10.1093/pch/pxz016 PMC6438864 30948917

[11] Graham ID, Logan J, Harrison MB, et al Lost in knowledge translation: Time for a map? J Contin Educ Health Prof 2006;26(1):13–24. 16557505 10.1002/chp.47

[12] Damschroder LJ, Aron DC, Keith RE, Kirsh SR, Alexander JA, Lowery JC Fostering implementation of health services research findings into practice: A consolidated framework for advancing implementation science. Implement Sci 2009;4:50. 19664226 10.1186/1748-5908-4-50 PMC2736161

[13] Gagliardi AR, Berta W, Kothari A, Boyko J, Urquhart R Integrated knowledge translation (IKT) in health care: A scoping review. Implement Sci 2016;11:38. 26988000 10.1186/s13012-016-0399-1 PMC4797171

[14] Kikuta A, Gardezi F, Dubey V, Taddio A Practices and perceptions about pain and pain management during routine childhood immunizations: Findings from a focus-group study with nurses working in Toronto Public Health. CJIDMM 2011;22:1–6. 10.1155/2011/381864 PMC3142592 22654924

[15] Taddio A, Ipp M, Thivakaran S, et al Survey of the prevalence of immunization non-compliance due to needle fears in children and adults. Vaccine 2012;30(32):4807–12. 22617633 10.1016/j.vaccine.2012.05.011

[16] Freedman T, Taddio A, McMurtry CM, et al; the Pain Pain Go Away Team Involving stakeholders in informing development of a Knowledge Translation (KT) intervention to improve the vaccination experience at school. Paediatr Child Health 2019. doi:10.1093/pch/pxz017 PMC6438867 30948919

[17] Taddio A, Freedman T, Wong H, et al; the Pain Pain Go Away Team Stakeholder feedback on The CARD™ system to improve the vaccination experience at school. Paediatr Child Health 2019. doi:10.1093/pch/pxz018 PMC6438868 30948920

[18] Taddio A, Ilersich ANT, Ilersich ALT, et al; the Pain Pain Go Away Team Piloting The CARD™ System for education of students about vaccination: Does it improve the vaccination experience at school? Paediatr Child Health 2019. doi:10.1093/pch/pxz019 PMC6438862 30948921

[19] Freedman T, Taddio A, Alderman L, et al; the Pain Pain Go Away Team. The CARD™ system for improving the vaccination experience at school: Results of a small-scale implementation project on student symptoms. Paediatr Child Health 2019. doi:10.1093/pch/pxz020 PMC6438866 30948922

[20] Taddio A, Alderman L, Freedman T, et al; the Pain Pain Go Away Team The CARD™ system for improving the vaccination experience at school: Results of a small-scale implementation project on program delivery. Paediatr Child Health 2019. doi:10.1093/pch/pxz021 PMC6438865 30948923

[21] Marfurt-Russenberger K, Axelin A, Kesselring A, Franck LS, Cignacco E The experiences of professionals regarding involvement of parents in neonatal pain management. J Obstet Gynecol Neonatal Nurs 2016;45(5):671–83. 10.1016/j.jogn.2016.04.011 27497029

[22] Paling J Strategies to help patients understand risks. BMJ 2003;327(7417):745–8. 14512489 10.1136/bmj.327.7417.745 PMC200818

[23] Institute of Medicine (US) Committee on Quality of Health Care in America. Crossing the Quality Chasm: A New Health System for the 21st Century. Washington, DC: National Academies Press (US); 2001 2, Improving the 21st-century Health Care System. <https://www.ncbi.nlm.nih.gov/books/NBK222265/> (Accessed January 19, 2019). 25057539

[24] Hart RA 1992 Children’s participation, from tokenism to citizenship. Florence: UNICEF <https://www.unicef-irc.org/publications/pdf/childrens_participation.pdf> (Accessed January 19, 2019).

[25] Ministry of Education. Growing Success: Assessment, Evaluation and Reporting in Ontario Schools, 1st edition, Covering Grades 1 to 12. 2010. <http://www.edu.gov.on.ca/eng/policyfunding/growSuccess.pdf> (Accessed January 19, 2019).

