# Supplementary figures and images for: Overview of a Knowledge Translation (KT) Project to improve the vaccination experience at school: The CARD™ System
Source: Paediatr Child Health. 2019 Mar 29;24(Suppl 1):S3–S18. doi: 10.1093/pch/pxz025 (PMC6438869; doi:10.1093/pch/pxz025)

# Cause and Effect Diagram

## Niagara Region Public Health School Vaccinations

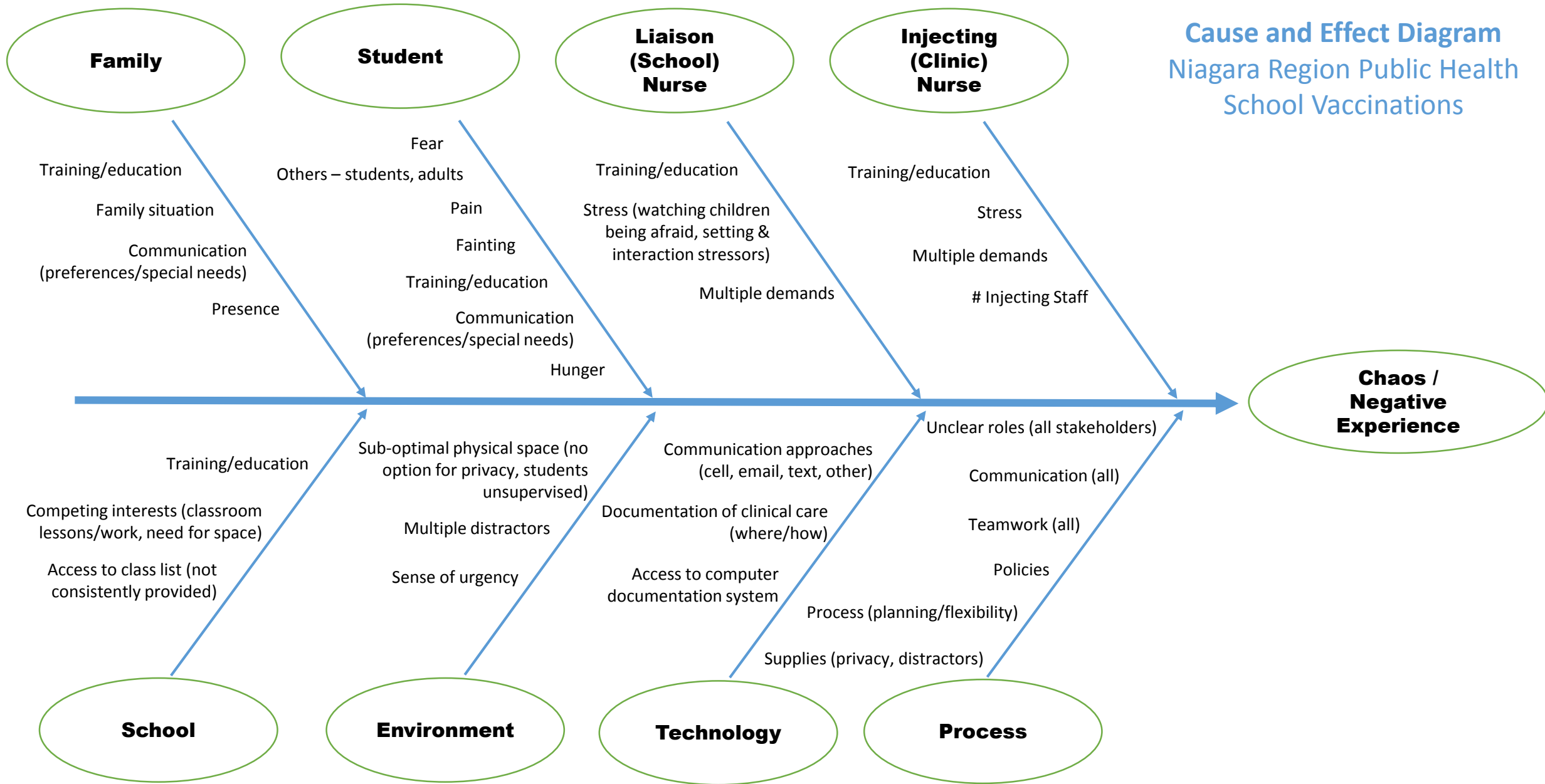

Supplement: Supplementary Figure 1 [file pxz025_suppl_supplementary_figure_1.pdf]
