# Supplementary material for: Overview of a Knowledge Translation (KT) Project to improve the vaccination experience at school: The CARD™ System
Source: Paediatr Child Health. 2019 Mar 29;24(Suppl 1):S3–S18. doi: 10.1093/pch/pxz025 (PMC6438869; doi:10.1093/pch/pxz025)
Supplement: Supplementary Figure 2 [file pxz025_suppl_supplementary_figure_2.pdf]

C

COMFORT

A

ASK

R

RELAX

D

DISTRACT

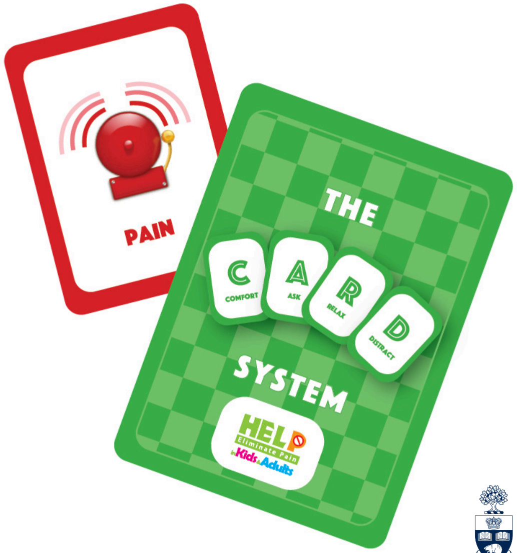

THE CARD SYSTEM

These four strategies will help you with your vaccination. Use the suggestions on the back to fill in the cards. Cut them out and carry them with you so you can remind yourself wherever you are.

CIHR IRSC
Canadian Institutes of Health Research
Instituts de recherche en santé du Canada

Immunize Immunisation Canada
immunize.ca

HELP
Eliminate Pain
in Kids & Adults

UNIVERSITY OF TORONTO
LESLIE DAN FACULTY OF PHARMACY

Niagara Region

Ontario

aboutkidshealth
Trusted answers from The Hospital for Sick Children

SickKids®

Cut here.

WHAT DISTRACTIONS WILL YOU USE?

____

____

____

____

____

____

WHAT WILL YOU DO TO RELAX?

____

____

____

____

____

____

WHAT WILL YOU ASK?

____

____

____

____

____

____

HOW WILL YOU BE COMFORTABLE?

____

____

____

____

____

____

HOW TO DISTRACT YOURSELF

Talk to someone.

Play video games.

Read books.

Play music.

Rub your arm.

Sing.

Allow yourself to daydream.

HOW TO RELAX

Do belly breathing (pretend to blow out a candle).

Do some self-talk (tell yourself you can handle this).

Have a friend with you.

Have a family member or trusted adult with you.

Have privacy.

QUESTIONS TO ASK

What will happen on my turn?

What vaccine am I getting?

Can I ...

- get the vaccine in a private room?
- use numbing creams or patches?
- bring my friend?
- bring a family member?
- bring a trusted adult?
- look at the needle?

HOW TO GET COMFORTABLE

Wear short sleeves, or something that lets you show your upper arm easily for the needle.

Eat a snack.

Bring a favourite item.

Sit up in a chair.

Make your arm loose or jiggly (like cooked spaghetti).

Tense your stomach and leg muscles if you get dizzy.
