# Supplementary material for: Overview of a Knowledge Translation (KT) Project to improve the vaccination experience at school: The CARD™ System
Source: Paediatr Child Health. 2019 Mar 29;24(Suppl 1):S3–S18. doi: 10.1093/pch/pxz025 (PMC6438869; doi:10.1093/pch/pxz025)
Supplement: Supplementary Figure 3 [file pxz025_suppl_supplementary_figure_3.pdf]

Preparing for vaccinations at school:

A guide for school staff

Vaccines are medicines that teach the body to recognize germs that cause diseases. If the body comes in contact with the germs in the future, it will be able to stop them.

Most vaccines are given with a needle. This can be painful or scary for some children and may discourage them from getting vaccinated. Use this factsheet to help prepare students for a vaccination at school.

Before clinic day

- Teachers attend student education sessions by the school nurse and reinforce messages.
- Teachers help with the completion, return and safe storage of the signed consent form by the student and legal guardian.
- Teachers work with students and the school nurse to identify and accommodate student requests from the **CARD** system.
- Teachers practise the **CARD** system (**C**omfort, **A**sk, **R**elax, **D**istract) with students to help them cope during vaccination:

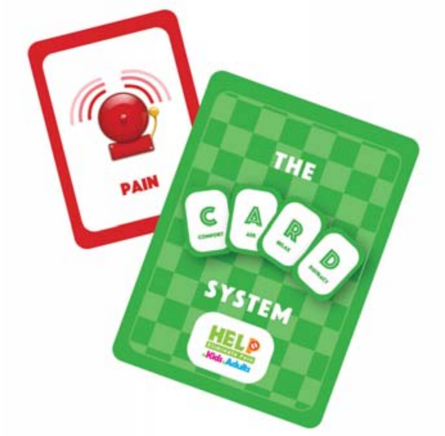

C

Comfort

- The student can ...
    - sit upright during the needle.
    - wear short sleeves, or something that lets them show their upper arm easily.
    - relax their arm.

A

Ask

- Ask the student about their preferences to make the needle more comfortable. For example, does the student want to ...
    - bring a friend or a trusted adult?
    - get the needle in a private room?
    - use numbing creams or patches? These are medicines that parents can buy at the pharmacy without a prescription. They dull the pain where the needle enters the skin. These take some time to work, so parents need to plan ahead.

R

Relax

- The student can take deep belly breaths before, during and after the needle. This is like blowing up a balloon or blowing out candles. The belly should move out when breathing in and move in when breathing out.

D

Distract

- The student can talk to someone or bring an object to get their mind off the needle (for example, music, game or book). Some students like to look at the needle – this is OK too. The student can let the nurse know their preferences.

On clinic day

- Allow time for a snack before vaccination.
- Use the **CARD** system to make the needle more comfortable: Allow students to bring supplies they need to distract themselves, such as personal devices.
- Foster a calm environment. Work with the nurse to help children stay calm and ensure an orderly flow through the vaccination clinic.
- Identify for the nurse any students that have special requests (such as privacy or the company of a friend during vaccination).
- Monitor students when they return to class and send any students who are feeling unwell back to the nurse with a buddy.

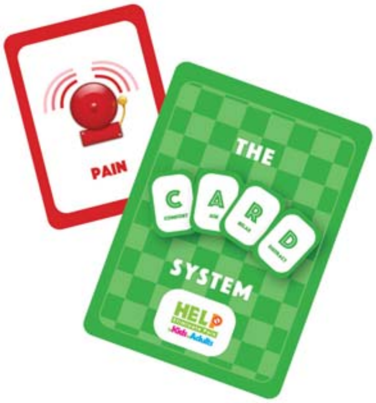

Serious adverse events following a vaccine

If a student experiences a serious adverse event after the nurse has left the school, follow school protocol (call emergency services or send student home with recommendation to contact family physician).

Some signs and symptoms of serious adverse events may include, but are not limited to:

- Flushed face, hives, and/or itching
- Swelling of the eyes, lips, face or tongue
- Difficulty breathing
- Dizziness and/or headache
- Nausea and/or vomiting

For more information, talk to your school nurse or contact:
Niagara Region Public Health Vaccine Preventable Disease Program
905-688-8248 ext. 7425
Toll free: 1-888-505-6074

CIHR IRSC Canadian Institutes of Health Research / Instituts de recherche en santé du Canada
Immunize Immunisation Canada immunize.ca
HELP Eliminate Pain in Kids & Adults
University of Toronto Leslie Dan Faculty of Pharmacy
Niagara Region
Ontario
