# Supplementary material for: Overview of a Knowledge Translation (KT) Project to improve the vaccination experience at school: The CARD™ System
Source: Paediatr Child Health. 2019 Mar 29;24(Suppl 1):S3–S18. doi: 10.1093/pch/pxz025 (PMC6438869; doi:10.1093/pch/pxz025)
Supplement: Supplementary Figure 4 [file pxz025_suppl_supplementary_figure_4.pdf]

Preparing children for school vaccinations:

A parent’s guide

Vaccinating your child at school is convenient and effective. Vaccines are medicines that teach the body to recognize germs that cause diseases. If the body comes in contact with the germs in the future, it will be able to stop them.

Most vaccines are given with a needle. This can be painful or scary for some children and may discourage them from getting vaccinated. Use this factsheet to help prepare your child for a vaccination. It includes information about what to expect and how to be ready.

Talk to your child about...

- What will happen: “You will get a vaccine to keep you healthy. The vaccine goes in your arm with a needle.”
- How the needle will feel: “There may be a pinch, some pushing or pressure for a few seconds. It bothers some kids, but others think it is ok.”
- How your child can increase comfort using the **CARD** system (**C**omfort, **A**sk, **R**elax, **D**istract): “The nurse will do some things so that the needle doesn’t bother you. You can do some things too. Use the **CARD** system. We can practise at home.”

The CARD system:

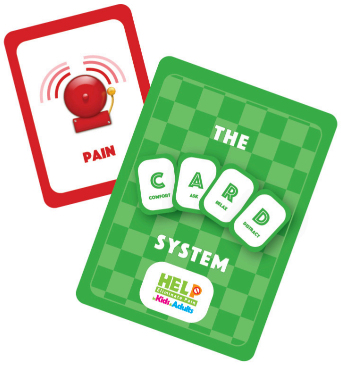

C

Comfort

- Your child can sit upright during the needle and relax the arm.

A

Ask

- Ask your child how to make the needle more comfortable. For example, does your child want to ...
    - bring a friend or a trusted adult with them?
    - get the needle in a private room?
    - use numbing creams or patches? These are medicines that parents can buy at the pharmacy without a prescription. They dull the pain where the needle enters the skin. They take some time to work, so plan ahead. They’re safe for all ages.

R

Relax

- Your child can take deep belly breaths before, during and after the needle. This is like blowing up a balloon or blowing out candles. The belly should move out when breathing in and move in when breathing out.

D

Distract

- Your child can talk to someone or bring an object to get their mind off the needle (for example, music, game or book). Some children like to look at the needle – this is OK too. Your child can let the nurse know his or her preferences.

Special tip for children who get dizzy or faint during needles

Your child can sit and tense their leg and stomach muscles until they feel warm in the face like they are blushing (10-15 seconds). Then they can release the muscle back to normal for 20-30 seconds. Repeat 5 times. Do not tense the arm where the needle goes in - keep it loose and jiggly.

Prepare your child...

- Sign the permission form with your child and return it to the school. Your child will be asked to agree again on the day of vaccination.
- Work with your child, public health department and school if you have any questions or if you are using strategies from the **CARD** system that require planning (such as privacy, numbing creams or patches).

Tips for your child on vaccination day...

- Try to eat something before vaccination.
- Wear short sleeves or something easy to pull up so that the upper arm can be reached easily.
- Use the **CARD** system to make needles more comfortable: **C**omfort, **A**sk, **R**elax, **D**istract. Bring any supplies you need, such as something to distract the mind or numbing cream.
- Be calm and positive and help other children who might be nervous.

What can you expect after the needle?

- Sometimes there are minor side effects like pain, swelling or redness of the arm. This is normal and does not last very long (a few minutes to a few days). It should not prevent your child from doing any activities.
- If you notice a change in your child's health that worries you, contact your healthcare provider or public health department.

For more information , talk to your school nurse or contact:
Niagara Region Public Health Vaccine Preventable Disease Program
905-688-8248 ext. 7425
Toll free: 1-888-505-6074

CIHR IRSC
Canadian Institutes of Health Research
Instituts de recherche en santé du Canada

Immunize Immunisation Canada
immunize.ca

HELP Eliminate Pain in Kids & Adults

UNIVERSITY OF TORONTO
LESLIE DAN FACULTY OF PHARMACY

Niagara Region

Ontario

aboutkidshealth
Trusted answers from The Hospital for Sick Children

SickKids
