# Supplementary material for: Overview of a Knowledge Translation (KT) Project to improve the vaccination experience at school: The CARD™ System
Source: Paediatr Child Health. 2019 Mar 29;24(Suppl 1):S3–S18. doi: 10.1093/pch/pxz025 (PMC6438869; doi:10.1093/pch/pxz025)
Supplement: Supplementary Figure 5 [file pxz025_suppl_supplementary_figure_5.pdf]

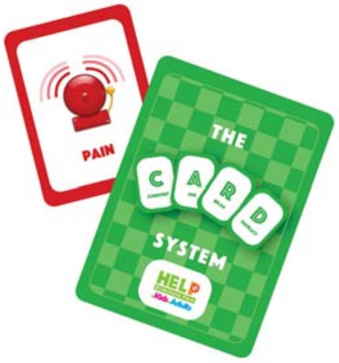

THE CARD SYSTEM

These four strategies can help you with your vaccination.

C	A	R	D
COMFORT	ASK	RELAX	DISTRACT

HOW TO GET COMFORTABLE

Wear short sleeves, or something that lets you show your upper arm easily for the needle.

Eat a snack.

Bring a favourite item.

Sit up in a chair.

Make your arm loose or jiggly (like cooked spaghetti).

Tense your stomach and leg muscles if you get dizzy.

QUESTIONS TO ASK

What will happen on my turn?

What vaccine am I getting?

Can I ...

- get the vaccine in a private room?
- use numbing creams or patches?
- bring my friend?
- bring a family member?
- bring a trusted adult?
- look at the needle?

HOW TO RELAX

Do belly breathing (pretend to blow out a candle).

Do some self-talk (tell yourself you can handle this).

Have a friend with you.

Have a family member or trusted adult with you.

Have privacy.

HOW TO DISTRACT YOURSELF

Talk to someone.

Play video games.

Read books.

Play music.

Rub your arm.

Sing.

Allow yourself to daydream.

HOW WILL YOU BE COMFORTABLE?

WHAT WILL YOU ASK?

WHAT WILL YOU DO TO RELAX?

WHAT DISTRACTIONS WILL YOU USE?

CIHR IRSC
Immunize Immunisation Canada immunize.ca
HELP Eliminate Pain in Kids & Adults
University of Toronto Leslie Dan Faculty of Pharmacy
Niagara Region
Ontario
aboutkidshealth
Trusted answers from The Hospital for Sick Children
SickKids
