# Supplementary material for: Overview of a Knowledge Translation (KT) Project to improve the vaccination experience at school: The CARD™ System
Source: Paediatr Child Health. 2019 Mar 29;24(Suppl 1):S3–S18. doi: 10.1093/pch/pxz025 (PMC6438869; doi:10.1093/pch/pxz025)
Supplement: Supplementary Figure 6 [file pxz025_suppl_supplementary_figure_6.pdf]

## *SAMPLE TEMPLATE* CHECKLIST FOR PUBLIC HEALTH FOR INTERVENTION SCHOOL CLINICS

**All items below are being tracked as part of planning, executing and fidelity checks. Associated time for key activities will also be tracked using public health usual time management processes.**

**School: ___________________________** **Phone: _______________________**
**Principal: _________________________** **Fax: _________________________**
**Contact Person: ___________________** **School Nurse:__________________**

| Initials | Procedures | Rd #1 | Rd #2 |
|---|---|---|---|
| 1. | **Clinic planning:**<br>Contacted principal via e-mail, phone call or face-to-face to organize information for immunization clinic (late August)<br>☐ Date confirmed for clinics:__________________________________________<br>☐ Date and time for immunization teaching (if not organized with teacher):____________________________, Date, time and person scheduled with:_____________________________________________<br>☐ Allowing 1 period or 2 periods?______________________________________<br>☐ Time of am recess:_______________________________________________<br>Time of lunch:____________________________________________________<br>☐ Room of clinic:__________________________________________________<br>Space/Room to accommodate privacy (this includes a space for the student to lay down if necessary, room for 3-4 individuals and no clear (see-through) walls/windows):_______________________________________________<br>☐ Principal agreed to have equipment available for clinic day (tables, chairs, gym mats).<br>☐ Space/room and how to accommodate students waiting:_________________<br>_______________________________________________________________<br>☐ Supplies available to occupy students while waiting:_____________________<br>_______________________________________________________________<br>☐ Reviewed educational materials with principal (e.g., videos, slides, pamphlets)?<br>☐ Distributed educational material to Principal for school staff and set up time for staff to review material (videos, other) – e.g., staff meeting, other. Date/time:_____________________________________________<br><br>Principal confirmed procedures to accommodate:<br>☐ electronic devices (If No, alternative – e.g., school ipads) **Yes/No**<br>☐ parent support<br>☐ peer support<br>☐ topical anesthetics<br>☐ food availability | | |

| | | | |
|---|---|---|---|
| | Principal agreed to place immunization teaching date and immunization clinic dates on:<br>☐ School calendar.<br>☐ Short newsletter insert.<br>☐ PA announcement to be made on the day prior to and on the day of clinic.<br>☐ Send e-mail blast to grade ___ students' families prior to clinic date to include reminders.<br>☐ Principal e-mail template provided.<br>☐ Principal stated he/she wishes staff can participate during clinic by:______________________________________________________<br>(e.g., identify students with special needs, practice and review CARD with students)<br>☐ Communicated with injecting charge nurse via e-mail.<br>☐ Parent support during clinic – organization and/or presence.<br>☐ Updated nurse's checklist and e-mailed to the vaccinating charge RN. Charted information on EMR/Profile. | | |
| **2.** | **Organized immunization teaching:**<br>☐ Contacted teacher(s) to organize date of immunization teaching (if not completed with principal).<br>Date/time:______________________________________<br>☐ Organized a date for a second visit after teaching for Q&A and check consent forms.<br>Date/time:_____________________________________________<br>☐ Reviewed with teacher:<br>□ Layout of presentation discussed.<br>□ Review materials with teacher prior to teaching.<br>□ Teacher states the number of students in classroom. (specify per class and total in school for applicable grades)<br>Grade __: #____________________<br>Grade __: #____________________<br>□ Teacher provided class list.<br>□ Teacher to change class activity for vaccination clinic day to a non-stressful activity (e.g., playing a movie, working on a fun activity etc.)<br>☐ Charted information on EMR/Profile. | | |
| **3.** | **In-class teaching:**<br>☐ All materials brought to teaching.<br>☐ Class teacher present during teaching.<br>(specify per class and total in school for all applicable grades)<br>Grade __: #________________<br>☐ Engaged in teaching. **Yes/No**<br>If **No**, explain:__________________________________________<br>Grade __: #________________<br>☐ Engaged in teaching. **Yes/No**.<br>If **No**, explain:_______________________________________<br>☐ Education provided to students about vaccination and CARD system.<br>☐ Number of students that received education? __________<br>Students that were absent? _____________ | | |

| | | | |
|---|---|---|---|
| | ☐ Case scenarios (practice) reviewed?<br>If NO, which did you review? ____________________<br>☐ Time allowed for Q&A.<br>☐ Questions by students regarding needle fear.<br>#______________<br>☐ CARD pamphlets filled in by students.<br>#______________<br>☐ Teacher reminded of return visit date for consent checking and extra Q&A period. (explain:_____________________________________________)<br>☐ Note made of which students were absent during teaching.<br>Subject number of absent students (for tracking purposes):<br>________________________________________<br>☐ Asked teacher to review information with students who are absent.<br>☐ Logged the "C", "A", "R," "D" requests by each student in the classroom to flag highly anxious students, topical anesthetic use, privacy, and friend requests.<br>☐ Logged self-identified and teacher-identified students who are anxious/worried about the upcoming school vaccinations.<br>☐ Communicated to injecting charge nurse post teaching.<br>☐ Charted information in EMR/Profile. | | |
| **4.** | **Consent Check/ Extra Q & A:**<br>☐ Revisited classroom to check consents.<br>Date:__________________<br>☐ Reviewed CARD with students absent from teaching.<br>☐ Reviewed case scenarios not reviewed in initial teaching.<br>☐ Secretary provided 2 class lists per class.<br>☐ Reviewed consents for blue/black pen, signatures from both student and parent/guardian, teacher's name, and that all boxes are filled out whether answer is "yes or no".<br>☐ Consents organized in alphabetical order.<br>☐ Worked with teacher to flag students who have anxiety, special needs, require privacy or peer support in regards to the vaccine.<br>☐ Flagged student consent forms of those who have anxiety about the vaccine.<br>☐ Extra Q&A provided for students?<br>☐ Follow up with teacher if they were able to provide vaccine information to absent students from in-class teaching.<br>☐ Reminded students about immunization clinic protocols and procedures, to wear a short sleeved shirt, eat breakfast prior to clinic, bring distraction aids and CARD.<br>☐ Remind principal to send out e-mail blast to parents.<br>☐ Provided template email to principal.<br>☐ Numbered consents with Subject number (for tracking purposes).<br>☐ Charted information in EMR/Profile. | | |
| **5.** | **Clinic Day setup**:<br>☐ Collected consent forms from classroom teacher to assist injecting charge nurse in organizing and allowing injecting nurse to make phone calls if necessary.<br>☐ Brought distraction kit to clinic.<br>☐ Brought 2 emergency kits to clinic to accommodate for private space. | | |

☐ Ensured food is available (e.g., food program, or faint kit).
Where is the food available? ________________________________________

☐ Ensured mats are available for main clinic and private room.

☐ Clinic room is same as what was planned with Principal. If not, specify: _____

☐ Separated clinic tables/workstations that are in main clinic space so that students are not too close to one another

☐ Set up tables so that clinic stations have children facing away from each other.

☐ Dispersed table dividers and distraction kit items among clinic stations.

☐ Put opaque covering (e.g., paper) for clinic door windows.

☐ Put sign on door to prevent interruptions (e.g., "library closed for vaccine clinic").

☐ When clinic was ready to commence, visited classroom(s) to introduce nursing staff, review clinic processes and CARD, answer last minute questions, remind students to bring any distraction aids, and confirm CARD choices.

☐ Worked with teacher to identify/confirm students with special requests (e.g., privacy, peer support, other).

☐ Organized students based on which should be accommodated for vaccines first based on CARD pamphlet request log (e.g., fearful students first, those students who require privacy, those students who have placed topical anesthetic etc.).

**School Staff involvement:**

☐ Discussed with school staff how they will assist the clinic.

☐ Roles of school staff include:

- ☐ Identifying students with special needs. **Yes/No**
- ☐ Sending students to and from the clinic. **Yes/No**
- ☐ Being available to support the students as needed. **Yes/No**
- ☐ Practice and review CARD system with students. **Yes/No**
- ☐ Minimize distress-promoting behaviours. **Yes/No**
- ☐ Minimize clinic interruptions. **Yes/No**

☐ Charted information in EMR/Profile.

**Vaccination procedure:**

☐ Supported the clinic by organizing the students, facilitated the flow of the clinic and monitored student's pre and post immunization, and supported nurses when required. (Students to return to classroom directly after immunization with partner, if in private- one nurse to escort student back to classroom).

☐ Managed and distracted students in waiting area (attempt to de-escalate nervous energy, dissuade inappropriate conversations among students).

☐ Asked each child to rate their fear on a 0-3 scale before immunizing.

☐ Asked each child which CARD strategies he/she would like to use.

☐ Asked students to fill in Student symptom survey with student symptoms.

☐ Filled out the Nurse checklist for each student with vaccination details.

☐ Charted students that returned to clinic because they were feeling unwell.

☐ Students returned to class in pairs after completing symptom survey.

☐ Nurses debriefed after end of clinic (specify who participated).
