# Supplementary material for: Overview of a Knowledge Translation (KT) Project to improve the vaccination experience at school: The CARD™ System
Source: Paediatr Child Health. 2019 Mar 29;24(Suppl 1):S3–S18. doi: 10.1093/pch/pxz025 (PMC6438869; doi:10.1093/pch/pxz025)
Supplement: Supplementary Appendix 1 [file pxz025_suppl_supplementary_appendix_1.pdf]

Tool Feedback Survey

Name of Tool: _______________________

1. Understood the information (circle): all of it, most of it, some of it, none of it

2. Amount of information (circle): too much, just right, not enough

3. What did you learn?____

4. How easy was it to use? ____

5. How you would use this tool (if at all): ____

6. How would you modify/change this tool (if at all): _______________________________

7. What would you add to it: ____

8. What would you take away from it: ____

(repeat for each tool reviewed)
