# Supplementary material for: Overview of a Knowledge Translation (KT) Project to improve the vaccination experience at school: The CARD™ System
Source: Paediatr Child Health. 2019 Mar 29;24(Suppl 1):S3–S18. doi: 10.1093/pch/pxz025 (PMC6438869; doi:10.1093/pch/pxz025)
Supplement: Supplementary Appendix 2 [file pxz025_suppl_supplementary_appendix_2.pdf]

Knowledge Questions*

The needle poke from vaccine injections can cause pain. Also, we know that some people are afraid or worried about the pain from needle pokes. Tell us if you think any of the ways described below can help to make needles more comfortable, either by making the needle poke hurt less, or by making it less scary. Put a checkmark (√) for “Yes” or “No” or “Don’t know” for each of the statements and explain your answer.

**Ways to make needles more comfortable**	**Yes**	**No**	**Don’t know**	**Explain why you think it works or doesn’t work**
Have someone with you like a parent or friend	☐	☐	☐	
Have privacy so people cannot see each other getting the needle	☐	☐	☐	
Use medicine to numb the skin so you don’t feel the needle	☐	☐	☐	
Distract yourself so you are paying attention to something else	☐	☐	☐	
Relax by taking deep belly breaths to help you stay calm	☐	☐	☐	
Ask questions so you know what will happen	☐	☐	☐	
Relax the arm getting the needle so that it is jiggly like spaghetti	☐	☐	☐	
Sit down in a comfortable position	☐	☐	☐	
Look away from the needle	☐	☐	☐	
Make your legs and tummy muscles tight (or tense) so you don’t feel dizzy	☐	☐	☐	

* Scores range from 0-10; responses to all questions are ‘true’
