# Supplementary material for: Overview of a Knowledge Translation (KT) Project to improve the vaccination experience at school: The CARD™ System
Source: Paediatr Child Health. 2019 Mar 29;24(Suppl 1):S3–S18. doi: 10.1093/pch/pxz025 (PMC6438869; doi:10.1093/pch/pxz025)
Supplement: Supplementary Appendix 3 [file pxz025_suppl_supplementary_appendix_3.docx]

**Your feedback is important to us!**

**Please tell us about your experience with school vaccinations today.**

1. **Tell us how much the needle hurt. If you had more than one needle, just tell us about how much it hurt overall. Pick a number from 0 to 10, where 0 is no pain and 10 is worst possible pain.**

0  1  2  3  4  5  6  7  8  9  10

1. **Tell us how scared you were about the needle. If you had more than one needle, just tell us about how scared you were overall. Pick a number from 0 to 10, where 0 is no fear and 10 is worst possible fear.**

0  1  2  3  4  5  6  7  8  9  10

1. **Tell us whether you were dizzy before, during or after the needle(s). Pick a number from 0 to 10, where 0 is not dizzy at all and 10 is most dizzy possible.**

0  1  2  3  4  5  6  7  8  9  10

1. **Do you have any suggestions for how to make school vaccinations a better experience for you next time?**

­­­­­­­­­­­­_________________________________________________________________________

­­­­­­­­­­­­­­­­_________________________________________________________________________
