# Supplementary material for: Overview of a Knowledge Translation (KT) Project to improve the vaccination experience at school: The CARD™ System
Source: Paediatr Child Health. 2019 Mar 29;24(Suppl 1):S3–S18. doi: 10.1093/pch/pxz025 (PMC6438869; doi:10.1093/pch/pxz025)
Supplement: Supplementary Appendix 6 [file pxz025_suppl_supplementary_appendix_6.docx]

***Process Issues Documentation Checklist***

Location: _____________________________________________________________________

**Pre-Clinic Teaching and Consent Process (Round 1 only):**

Date of teaching *(dd/mm/yyyy)*: _______________ Time of day *(hr:min-hr:min)*: _______________

**# students in class**: class 1_________class 2_________ class 3 _________ N/A
**# students present**: class 1_________class 2_________class 3 _________ N/A

**Consent forms checked? Date** *(dd/mm/yyyy)*:_________________

# consent forms returned: _________

# phone calls made: _________ 🡪 Date *(dd/mm/yyyy)*:________________

Able to use class list to determine missing consents? Yes No

Reminders to return consents *(general to class, specific to student, robocalls to all)*:

______________________________________________________________________________________________________________________________________________________________________

Who was involved in providing these reminders *(principal, secretary, teachers, etc.)*?
Please specify.

______________________________________________________________________________________________________________________________________________________________________

***Process Issues Documentation Checklist – ALL ROUNDS***

Location: _____________________________________________________________________

Date of clinic *(dd/mm/yyyy)*: _______________

Time of day for arrival *(hr:min-hr:min)*: _______________

Time of day for departure *(hr:min-hr:min)*: _______________

**Set-up of room:** *Draw a basic diagram of the* ***vaccination clinic room and waiting area set-up****, including: tables, entrances/exits, bookcases/barriers, windows, and childrens’ and nurses’ positions.*

***Process Issues Documentation Checklist – ALL ROUNDS***

# Injecting Nurses (excluding Charge Nurse): ______

Did the Charge Nurse also give injections?  Yes.  No.

# Gr. 7 classes: ______

Was a class list used?  Yes.  No.  List was unavailable.

Describe the process used to retrieve students from their classroom.

______________________________________________________________________________________________________________________________________________________________________

Describe the process used to make students wait for their turn to be vaccinated.

______________________________________________________________________________________________________________________________________________________________________

Describe the process used to return students to their classroom.

______________________________________________________________________________________________________________________________________________________________________

Was the school nurse present?  Yes  No If yes, what was her role?

______________________________________________________________________________________________________________________________________________________________________

Were there any setting-specific or over-arching factors/characteristics of the vaccination day?  *E.g.: Special location or timing for vaccinations of fearful children.*

______________________________________________________________________________________________________________________________________________________________________

***Process Issues Documentation Checklist – ALL ROUNDS***

Document if the time needed to deliver vaccinations was impacted by any of the following issues.

| **Issue** | **Occurred?** | **Number if Occurred and Comments** |
| --- | --- | --- |
| School breaks (recess, lunch) | Yes  No |  |
| Record analysis | Yes  No |  |
| Option sheets (for absences) | Yes  No |  |
| Phone calls | Yes  No |  |
| Panorama (computer) issue | Yes  No |  |
| Cold chain | Yes  No |  |
| Double needle sticks | Yes  No |  |
| Faint | Yes  No |  |
| Anaphylaxis | Yes  No |  |
| Highly fearful children | Yes  No |  |
| CHILDREN RETURNING TO NURSE BECAUSE: | | |
| … feeling ill |  |  |
| … feeling faint |  |  |
| … have a headache |  |  |
| … want ice |  |  |
| …other (specify below) |  |  |

| **Vaccine** | **Number of Doses Given** |
| --- | --- |
| Hepatitis B |  |
| Meningococcal |  |
| Human Papilloma Virus |  |
| Other (specify): |  |

Document the number of doses of each vaccine administered at the clinic.
